# A comparative study of fifteen cover crop species for orchard soil management: water uptake, root density traits and soil aggregate stability

**DOI:** 10.1038/s41598-023-27915-7

**Published:** 2023-01-13

**Authors:** Caterina Capri, Matteo Gatti, Andrea Fiorini, Federico Ardenti, Vincenzo Tabaglio, Stefano Poni

**Affiliations:** Department of Sustainable Crop Production, Università Cattolica del Sacro Cuore, Via Emilia Parmense 84, 29122 Piacenza, Italy

**Keywords:** Grassland ecology, Plant physiology, Climate-change ecology, Plant ecology, Climate-change mitigation

## Abstract

Increasing the use of cover crops (CCs) is a necessity in sustainable viticulture, although it might clash with possible excessive competition towards vines. Especially in a climate-change scenario, the latter feature should be minimized while maintaining ecosystem services. Aimed at identifying CCs for vineyard floor management, the trial characterized several species according to their evapotranspiration (ET) rates, root growth patterns, and soil aggregate stability potential. The study was performed in 2020 in Piacenza (Northern Italy) on 15 CC species grown in pots kept outdoor and classified as grasses (GR), legumes (LE) and creeping (CR). Together with bare soil (control), they were arranged in a complete randomized block design. CCs ET was assessed through a gravimetric method, starting before mowing and then repeated 2, 8, 17 and 25 days thereafter. Above-ground dry biomass (ADW), root length density (RLD), root dry weight (RDW) and root diameter class length (DCL) were measured, and mean weight diameter (MWD) was calculated within 0–20 cm depth. Before mowing, ET was the highest in LE (18.6 mm day^−1^) and the lowest in CR (8.1 mm day^−1^) the latter being even lower than the control (8.5 mm day^−1^). The high ET rates shown by LE were mainly related to very fast development after sowing, rather than to a higher transpiration per unit of leaf area. After mowing, the 15 species’ ET reduction (%) plotted vs leaf area index (LAI, m^2^ m^−2^) yielded a very close fit (R^2^ = 0.94), suggesting that (i) a linear decrease in water use is expected anytime starting with an initial LAI of 5–6, (ii) a saturation effect seems to be reached beyond this limit. Selection of cover crop species to be used in the vineyard was mainly based on diurnal and seasonal water use rates as well as dynamic and extent of root growth patterns. Among GR, *Festuca ovina* stood out as the one with the lowest ET due to its “dwarfing” characteristics, making it suitable for a permanent inter-row covering. CR species confirmed their potential for under-vine grassing, assuring rapid soil coverage, lowest ET rates, and shallow root colonization.

## Introduction

Vineyards are frequently established on inherently poor soils^1^ and subjected to intensive management practices, threatening soil functions and associated ecosystem services^2–4^. Moreover, the Mediterranean climate is often characterized by severe summer droughts associated with short, yet heavy rainstorms in autumn-spring, favouring the run-off of surface waters^2,5^, soil degradation and erosion^6,7^. High surface water runoff due to short and heavy rainstorms in autumn-spring removes the more fertile topsoil layer, reducing soil organic matter (SOM) content and carbon (C) sequestration, nutrients availability and water-holding capacity leading to an overall decrease in soil fertility and crop productivity^8^. In addition, following SOM loss, soil aggregates tend to break down more easily and soil erodibility worsens^9,10^. Lastly, surface runoff and resulting soil erosion are the main routes through which fertilizer and pesticide residues reach surface waters^8^.

Conventional vineyard soil management affects soil properties^2,11^. Mechanical weeding may induce physical degradation of vineyard soils^7,12^, and modify soil biological communities at different trophic levels^13^. Conversely, vineyard cover cropping is considered a sustainable soil management strategy, as it boosts essential ecosystem services of soil^3^, including surface water infiltration^14^, C sequestration^15^, and reduced soil erosion^7,16,17^. Further, cover crops (CCs) can help to protect soil from water and/or wind erosion, as they improve soil aggregate stability^18^ and protect them from the raindrops impact^19^.

CCs can also help enhancing/maintaining a favourable soil structure and stable porosity in vineyards^20^ as root development and turnover directly influence subsoil structure, increasing macro-porosity. During growth, roots exert pressure which generates a reorganization of the soil pore network^21^. After root decomposition, root-dug channels remain empty, forming bio-pores^22,23^. Consequent to increased soil macro-porosity, soil surface hydraulic conductivity, water infiltration, and sub-soil refilling usually improve during the rainy season ^24,25^. During a rainfall event, if the soil becomes saturated, the hydraulic conductivity of the soil surface decreases, leading to surface water runoff^3^. Such a decrease is partly counteracted by the presence of a CC^26^. Further, CC leaf area reduces the kinetic energy of raindrops and promotes water infiltration as the staying time of water at the soil surface increases^24^.

The improved rainfall infiltration rate and enhanced soil water storage promoted by CCs might warrant additional soil water storage^25^. This is especially significant in areas where precipitation occurs over a relatively short time in a series of heavy rainfall events^3^, as in the Mediterranean area. However, vine growers in the Mediterranean regions are still quite reluctant to use CCs due to concerns about water and nutrient competition with the main crop^27,28^ as the above-mentioned additional water budget could be rapidly used (i.e. transpired), partly or totally by the CC itself^29^.

Typically, the most common technique of cover cropping involves the management of native species as readily available and inexpensive^4,30^ yet, usually being the most competitive for both water and nutrients^28,31^.

To mitigate or remove competition, CC is often terminated in spring with tillage^4^. Nonetheless, as a negative side effect of this decision, several benefits bound to the permanent cover of the vineyard soil (e.g. facilitated machine transit with wet soil, reduced soil erosion, etc.) are lost^4,32^. Therefore, identifying appropriate strategies (i.e. CC species and adoption of the best cultural practices) to maintain the permanent soil cover benefits, while reducing CC competition in vineyards, is still necessary.

According to the literature, mowing can be used as a useful short-term water preservation strategy^28,33^. After mowing, sward residual mass left in situ further protects the soil from erosion and runoff^34,35^, and improves soil health in the short term^36^, while reducing water competition and soil evaporation^33,37^.

To exploit as many positive externalities as possible and to reduce the potential problems associated with the presence of CC in a vineyard, it is advisable to switch from the use of native species to sown (i.e. selected) ones^30^. Moreover, when a high risk of water competition towards the consociated vine is assessed, the selection of the appropriate type of CCs becomes crucial, favouring those featuring reduced above-ground biomass and root development^30^, assuming such characteristics to be conducive to a lower water consumption^31,38^.

Unfortunately, to date, the winegrowers’ demand for low-competitive species is still largely unmet^38^. Agroscope (Changins—Wädenswil, Switzerland) has initiated the selection and propagation of low-competition genotypes for use in vineyards^39,40^. Moreover, the desirable ideotype should possess some other important characteristics: (i) good establishment capacity and resistance to repeated trampling; (ii) homogeneity and long-lasting soil cover; (iii) effective weed control; (iv) perennial habitus (to reduce seeding cost); (v) reduced aerial development (to reduce maintenance and vineyard interventions) and (vi) summer growth lag followed by autumn recovery.

Among almost fifty species, tested best results were obtained with *Hordeum murinum* and, to a less extent, *Trifolium subterraneum* and *Trifolium repens*^39^. Other studies have shown that perennial species (e.g. *Trifolium repens*) tend to be more competitive with vines, compared to annuals with spontaneous reseeding^40^. Not surprisingly, the less competitive ones are also those with greater difficulty in ensuring the establishment of the sward and maintaining a good soil coverage over time, often being invaded by native grass within two or three seasons^41^.

This pot trial is, to the best of our knowledge, the first case of a comparative screening to evaluate water use, root characterization, and anti-erosion potential of a large number of herbaceous species potentially targeted for vineyard use. Together with some already used CCs, such as grasses (GR) and legumes (LE), new creeping (CR) ones were included in the study for their potential interest as living mulches under the trellis.

The present study aimed to compare different CC species for (i) assessing water loss (use) before and after mowing, (ii) characterize root traits and clarify their effects on soil aggregation, and (iii) identify the most recommended species for vineyard cover cropping.

## Results

### Evapotranspiration measurements and above-ground biomass

**Figure 1:**
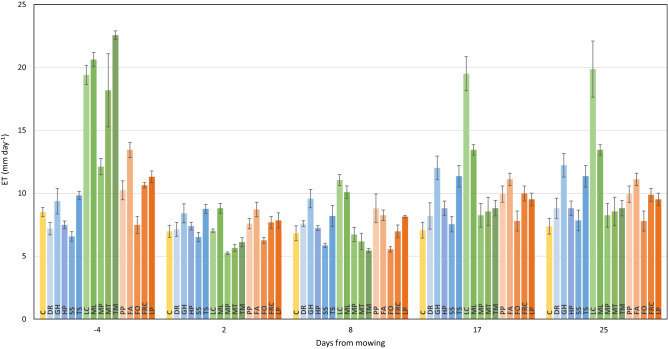
Vertical bars represent the daily water use as referred to unit of soil (ET, mm day^−1^) for the bare soil (yellow) and all the cover crop species as divided into creeping plants (shades of blue), legumes (shades of green) and grasses (shades of orange). Evapotranspiration was measured though a gravimetric method before (i.e. − 4) and at 2, 8, 17 and 25 days after mowing. ET data are mean values ± SE (n = 4).

Figure 1 shows daily evapotranspiration (ET, mm day^−1^) of each CC tested before mowing (DOY, day of the year, 184) and at 2, 8, 17 and 25 days after mowing (DOY 190, 196, 205 and 213); bare soil was also included as a reference. Before mowing, ET rates showed significant differences between and within the three groups. CR plants had a mean ET of 8.1 mm day^−1^, which was lower, compared to the other two groups (10.6 and 18.6 mm day^−1^ for GR and LE, respectively) and the bare soil control (8.5 mm day^−1^). On DOY 184, values as high as 9.4 (*Glechoma hederacea* L., GH) and 9.8 mm day^−1^ (*Trifolium subterraneum* L. cv. Denmark, TS) were found (Fig. 1), while ranging around 7 mm day^-1^, *Dichondra repens* J.R.Forst. & G.Forst. (DR), *Hieracium pilosella* L. (HP), and *Sagina subulata* (Swartz) C. Presl (SS) ET were lower than soil evaporation itself.

On the same day, a large ET variation was recorded within the GR group as *Festuca arundinacea* Schreb. cv. Thor (FA) scored the highest daily ET values (13.4 mm day^−1^), whereas in *Festuca ovina* L. cv. Ridu (FO), water loss was reduced by 45% (7.5 mm day^−1^). Within the 15 CCs, LE registered the highest pre-mowing ET with *Trifolium michelianum* Savi cv. Bolta (TM) peaking at 22.6 mm day^−1^. However, within LE, *Medicago polymorpha* L. cv. Scimitar (MP) showed ET values as low as 12.1 mm day^−1^ (Fig. 1).

Two days after mowing, all tested CCs recorded ET values lower than 9 mm day^−1^ (Fig. 1). Moreover, water use reduction among LE ranged between 56% (*M. polymorpha,* MP) and 73% (*T. michelianum*, TM), such that *T. michelianum* (TM, 6.1 mm day^−1^), *Medicago truncatula* Gaertn. cv. Paraggio (MT, 5.6 mm day^−1^) and *M. polymorpha* (MP, 5.2 mm day^−1^) registered ET values lower than the bare soil (7.0 mm day^−1^). Even though registering a consistent ET reduction after mowing, GR retained ET rates slightly higher than bare soil, except for *F. ovina* (FO), which recorded the lowest at 6.3 mm day^−1^. Subsequent samplings showed that most of the CCs had a progressive recovery in water use (Fig. 1) and data taken 17 days after mowing confirmed that *Lotus corniculatus* L. cv. Leo (LC) and all GR fetched pre-mowing ET rates. *Medicago lupulina* L. cv. Virgo (ML) registered a partial recovery with similar rates (about 13 mm day^−1^) at 17 and 25 days after the mowing event. *F. ovina* and all remaining LE stayed below 10 mm day^−1^ with ET values close to the control until the end of the trial. At 17 days from grass cutting, under a quite high exceeding-the-pot biomass, both *G. hederacea* (GH) and *T. subterraneum* (TS) reached ET values as high as 12.0 and 11.4 mm day^−1^, respectively. On the other hand, *D. repens* (DR), *H. pilosella* (HP), and *S. subulata* (SS) even though with slightly higher ET values than those registered at the beginning of the trial (DOY 184), remained close to the soil evaporation rates until DOY 213.

Aboveground dry clipped biomass at the first mowing date (ADW_MW1, DOY 188) showed large differences among groups, as represented in Table 1. ADW_MW1 within LE was quite variable, as values ranged between 274.3 g m^−2^ (*M. polymorpha,* MP) and 750.0 g m^−2^ (*T. michelianum*, TM). With a mean value of 565.9 g m^−2^, LE aboveground biomass was 80% higher than the mean GR ADW_MW1 (110.2 g m^-2^). *F. ovina* (FO) scored the lowest value at 48.4 g m^−2^ among grasses, while within the creeping group, *G. hederacea* (GH) and *T. subterraneum* (TS) had biomass development outside the pot edges totalling 89.6 g m^−2^ and 23.2 g m^−2^, respectively.

**Table 1:** Aboveground dry biomass clipped at the first mowing event (ADW _MW1), the corresponding leaf area surface index (LAI) and water use per leaf area unit (ET_LEAF_) of all cover crops tested.

Cover crop group	Treatment (T)	ADW_MW1 (g m^−2^)	LAI (m^2^ m^−2^)	ET_LEAF_ (mm m^−2^ day ^−1^)
Legumes	*Trifolium michelianum*	750.0a	12.4a	1.81d
	*Medicago polymorpha*	274.3c	2.5c	4.92bc
	*Medicago lupulina*	503.3b	5.1b	4.05bcd
	*Medicago truncatula*	641.2a	5.4b	3.40bcd
	*Lotus corniculatus*	660.7a	5.3b	3.65cd
Grasses	*Festuca arundinacea*	161.8cd	1.5cd	8.83a
	*Festuca ovina*	48.4d	1.0cd	7.75a
	*Festuca rubra commutata*	125.3cd	1.2cd	8.54a
	*Poa pratensis*	108.6cd	1.3cd	8.12a
	*Lolium perenne*	106.8cd	1.2cd	9.22a
Creeping	*Glecoma hederacea*	89.6d	0.8cd	3.68*bcd
	*Hieracium pilosella*	0.0d	–	3.86*bcd
	*Dichondra repens*	0.0d	–	5.46*b
	*Sagina subulata*	0.0d	–	–
	*Trifolium subterraneum*	23.2d	0.2d	2.74*bcd

Lowercase letters indicate significant differences among treatments (SNK test, p < 0.05) and *indicates ET_LEAF_ based on LAI estimated through photo analysis as creeping plants were not mowed.

Leaf area index (LAI, m^2^ m^−2^) at mowing showed the highest values in LE with LAI peaking at 12.4 (Table 1). Among GR, LAI did not show significant differences, being around 1.2. Concerning CR, LAI was assessed at 0.2 and 0.8 for *T. subterraneum* (TS) and *G. hederacea* (GH) respectively, while LAI estimated through photo analysis ranged between 1.3 (*D. repens,* DR) and 3.6 (*T. subterraneum* TS).

Evapotranspiration per leaf area unit (ET_LEAF_) was notably higher in GR, ranging between 7.75 (*F. ovina*, FO) and 9.22 (*Lolium perenne* L. cv. Playfast, LP) mm m^−2^ day^−1^ (Table 1). In descending order, ET_LEAF_ was the highest in *D. repens* (DR, 5.46 mm m^−2^ day^−1^). Similar ET_LEAF_ was found when comparing some LE and CR species such as *M. truncatula* (MT, 3.40 mm m^−2^ day^−1^), *M. lupulina* (ML, 4.05 mm m^−2^ day^−1^), *G. hederacea* (GH, 3.68 mm m^−2^ day^−1^), *H. pilosella* (HP, 3.86 mm m^-2^ day^-1^) and *T. subterraneum* (TS, 2.74 mm m^−2^ day^−1^). *T. michelianum* (TM), with 1.81 mm m^-2^ day^-1^ scored the lowest ET_LEAF_ of all species (Table 1).

**Figure 2:**
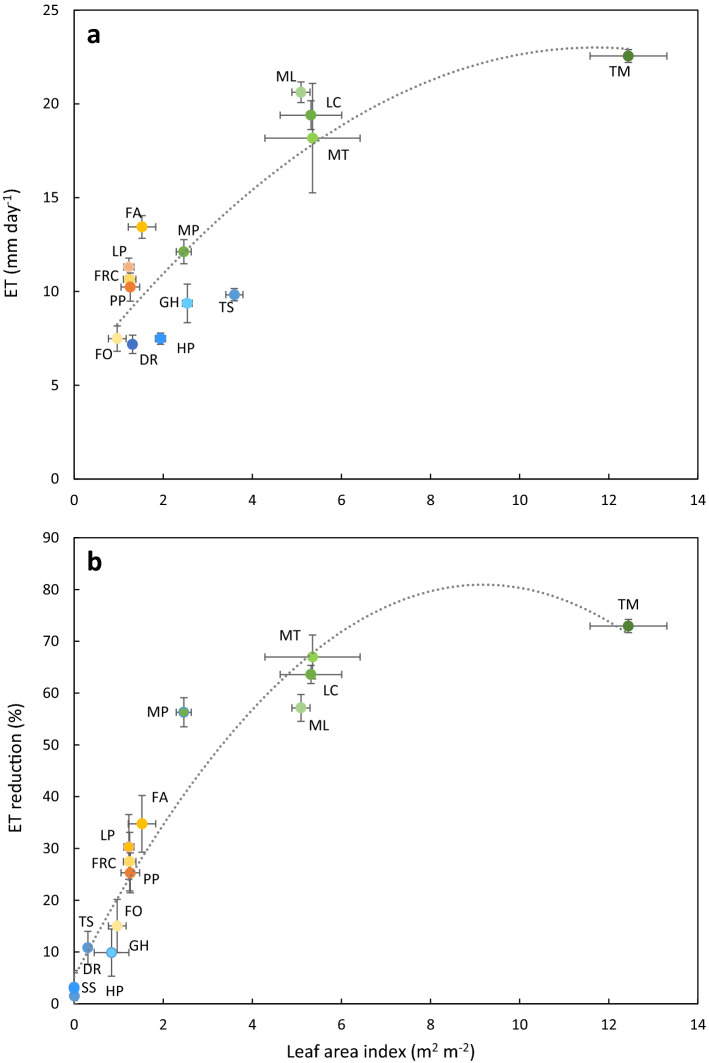
Panel (**a**): quadratic regression of leaf area index (LAI, m^2^ m^−2^) vs cover crop evapotranspiration per unit of soil (ET, mm day^−1^). Each data point is mean value ± SE (n = 4). The quadratic model equation is y = − 0.128x^2^ + 2.9968x + 5.4716, R^2^ = 0.76. Panel (**b**): the quadratic regression between LAI corresponding to the clipped biomass (m^2^ m^−2^) and cover crop ET reduction (%). Each data point is mean value ± SE (n = 4). Quadratic model equation is y = − 0.8985x^2^ + 16.503x + 5.1491, R^2^ = 0.94.

Plotting LAI versus the before-mowing ET yielded a significant quadratic relationship (R^2^ > 0.76) (Fig. 2a) which helped to distinguish two different data clouds. Till LAI values of about 6, the model was linear, having at its lower end all GR and CR species with the inclusion of *M. polymorpha* (MP) as a legume, while, at the other end, *M. truncatula* (MT), *L. corniculatus* (LC) and *M. lupulina* (ML) were grouped together. *T. michelianum* (TM) was isolated from all CCs at 22.56 mm day^−1^.

When regressing the fraction of ET reduction, compared to pre-mowing values vs LAI (Fig. 2b), the same quadratic model achieved a very close fit (R^2^ = 0.94, p < 0.01). CC grouping was similar to the patterns highlighted for ET, although more accurate predictions were reached at LAI, varying from 0 to 3. A linear ET reduction was shown when LAI removed through trimming ranged between 0 and 6, while thereafter, ET reduction was less than proportionate to the amount of LAI removed. This suggests an LAI of 5–6 as a benchmark, within which it is possible to maximise water use reduction after the trim.

### Root growth and soil colonization

Root length density (RLD, cm cm^-3^) determined for each CC at 0–10 cm and 10–20 cm depth is shown in Table 2. Within the topsoil layer, RLD of *Poa pratensis* L. cv. Tetris (PP), *Festuca rubra* L. var. *commutata* Gaud. cv. Casanova (FRC), and *F. arundinacea* (FA) peaked at 52.5; 53.7 and 59.0 cm cm^−3^, respectively, whereas *M. polymorpha,* (MP), *M. truncatula* (MT), *T. subterraneum* (TS) and *T. michelianum* (TM) did not reach the 10 cm cm^−3^ threshold (Table 2). *L. corniculatus* (LC) recorded the highest RLD (29.7 cm cm^−3^) at 0–10 cm among the LE species while being very close to *F. ovina* (FO, 30.3 cm cm^−3^), which had the lowest RLD within the GR group. In the CR group, the highest and lowest RLD values within the top layer were found in *G. hederacea* (GH) and *T. subterraneum* (TS), at 26.9 and 7.4 cm cm^−3^ respectively (Table 2). Looking at the root colonization of the 10–20 cm soil horizon, *F. arundinacea* maintained the highest RLD (10.7 cm cm^-3^), followed by *L. corniculatus* (7.9 cm cm^−3^). Overall, very low RLD was recorded through this layer in all the remaining CCs.

**Table 2:** Root length density (RLD) and diameter class length (DCL) for very fine (ø = 0–0.075 mm), fine (ø = 0.075–0.2 mm), medium (ø = 0.2–1 mm) and coarse (ø > 1 mm) root diameters as affected by soil cover.

Soil layer	Soil cover	RLD (cm cm^−3^)	DCL (cm cm^−3^)				RDW (mg ^cm−3^)
			Ø = 0.00–0.075 mm	Ø = 0.075–0.2 mm	Ø = 0.2–1.0 mm	Ø = > 1.0 mm	
0–10 cm	*Trifolium michelianum*	2.4g	0.619e	1.190f	0.537h	0.006de	3.7fg
	*Medicago polymorpha*	7.4fg	1.265de	4.170ef	1.843fgh	0.027de	4.0efg
	*Medicago lupulina*	13.6ef	1.486de	7.140de	4.738def	0.088bcde	4.8cdef
	*Medicago truncatula*	2.8g	0.290e	1.350f	1.078gh	0.008de	3.2g
	*Lotus corniculatus*	29.7cd	1.114de	4.870def	23.076a	0.540a	8.7a
	*Festuca arundinacea*	59.0a	17.637ab	25.740a	15.347b	0.216b	7.6ab
	*Festuca ovina*	30.3cd	9.749c	12.770bcd	7.384cde	0.068bcde	5.3cde
	*Festuca rubra commutata*	53.7ab	16.699ab	26.100a	10.769bc	0.071bcde	6.2bc
	*Poa pratensis*	52.5ab	23.354a	19.460ab	9.393c	0.178bc	6.1cd
	*Lolium perenne*	33.0bc	11.386bc	11.700bcd	9.844bc	0.032cde	5.1cdef
	*Dichondra repens*	16.1def	0.341e	7.550cde	8.025cde	0.107bcd	5.3cde
	*Trifolium subterraneum*	7.4fg	1.112de	4.080ef	2.195fgh	0.059bcde	4.2efg
	*Sagina subulata*	9.8fg	3.950d	2.960ef	2.667fg	0.001e	4.3efg
	*Glecoma hederacea*	26.9cde	1.736de	16.320abc	8.615cd	0.033cde	4.9cdef
	*Hieracium pilosella*	10.4fg	0.508e	5.540def	4.235ef	0.090bcde	4.7def
*P-value*		< 0.001	< 0.001	< 0.001	< 0.001	< 0.001	< 0.001
10–20 cm	*Trifolium michelianum*	0.2c	0.029c	0.074c	0.056cd	0.001c	2.3ab
	*Medicago polymorpha*	0.0c	0.025c	0.059c	0.016d	0.000c	0.8ab
	*Medicago lupulina*	1.6bc	0.295c	0.757bc	0.568cd	0.003bc	2.8ab
	*Medicago truncatula*	0.0c	0.000c	0.002c	0.002d	0.000c	0.0b
	*Lotus corniculatus*	7.9a	0.442c	1.297bc	6.173a	0.037a	4.5a
	*Festuca arundinacea*	10.7a	2.269a	5.215a	3.157b	0.016b	4.2a
	*Festuca ovina*	0.3c	0.068c	0.099c	0.090cd	0.000c	4.2a
	*Festuca rubra commutata*	1.3bc	0.249c	0.681bc	0.339cd	0.006bc	4.2a
	*Poa pratensis*	3.4b	1.369b	1.519b	0.521cd	0.008bc	3.7a
	*Lolium perenne*	2.4bc	0.605bc	0.824bc	1.008cd	0.000c	3.6a
	*Dichondra repens*	2.2bc	0.056c	0.732bc	1.415c	0.002bc	4.2a
	*Trifolium subterraneum*	0.1c	0.009c	0.039c	0.050cd	0.000c	1.7ab
	*Sagina subulata*	0.0c	0.000c	0.000c	0.000d	0.000c	0.0b
	*Glecoma hederacea*	0.5c	0.032c	0.228bc	0.220cd	0.000c	4.2a
	*Hieracium pilosella*	0.1c	0.004c	0.026c	0.068cd	0.000c	2.8ab
*P-value*		< 0.001	< 0.001	< 0.001	< 0.001	< 0.001	< 0.001

Lowercase letters indicate differences among treatments within the same soil layer. P-values are reported.

The highest values of diameter class length (DCL, mm cm^−3^) for very fine roots (DCL_VF, < 0.075 mm) in the first 10 cm soil were recorded in GR, ranging between 9.75 (*F. ovina,* FO) and 23.35 (*P. pratensis,* PP) cm cm^−3^ (Table 2). All remaining species recorded quite low values, comprised within the 0–4 cm cm^−3^ range. A similar pattern was observed in the same soil layer for the fine root class (DCL_F, 0.075–0.2 mm), although *F. arundinacea* (FA)and *F. rubra commutata* (FRC) scored the highest values (25.74 and 26.10 cm cm^−3^, respectively). For the same diameter class length, none among LE and CR exceeded the 9 cm cm^−3^ except for *G. hederacea*, assessed to be at 16.32 cm cm^−3^.

A more uniform behaviour among species was found for medium (DCL_M, 0.2–1.0 mm) and coarse (DCL_C, > 1.0 mm) roots although, most notably, *L. corniculatus* roots showed the highest abundance for both DCL_M (23.08 cm cm^−3^) and DCL_C (0.54 cm cm^−3^).

At the 10–20 cm soil depth, GR confirmed the highest values for both very fine and fine roots, with *F. arundinacea* reaching maximum DCL of 2.269 and 5.215 cm cm^-3^, respectively (Table 2). *L. corniculatus* largely outscored any other species for both medium and coarse root diameter (6.173 and 0.037 cm cm^−3^, respectively), with *F. arundinacea* ranking second (3.157 and 0.016 cm cm^−3^, respectively).

The highest root dry weight (RDW, mg cm^-3^) within the topsoil layer was reached by *L. corniculatus* (8.7 mg cm^−3^) and *F. arundinacea* (7.6 mg cm^-3^). Notably, such values were significantly higher than those recorded on the remaining species, except for the *F. arundinacea* vs *F. rubra commutata* comparison (Table 2). At 10–20 depth, scant variation was recorded in RDW measured in grasses, whereas *L. corniculatus* held its supremacy within legumes (4.5 mg cm^−3^). Within the creeping type, *D. repens* (DR) and *G. hederacea* (GH) scored RDW values as high as those determined for grass species (namely *F. arundinacea* , *P. pratensis* and *F. rubra commutata*), whereas *S. subulata* (SS) essentially had no root development.

### Soil aggregates and mean weight diameter (MWD)

Table 3 reports the proportional aggregate weight (g kg^−1^) for both 0–10 and 10–20 cm soil depths. Compared to bare soil, the largest increase in large macroaggregates (LM, > 2000 µm) in the top 10 cm of soil was achieved by *L. corniculatus* with 461 g kg^−1^. *L. corniculatus* differed from the rest of the LE group, whose grand mean (90 g kg^−1^) was the lowest of the three tested groups. As a legume, *T. subterraneum* (TS, 122 g kg^−1^) recorded the lowest values compared to fellow CR species, ranging between 211 (*D. repens,* DR) and 316 g kg^−1^ (*G. hederacea,* GH). GR recorded LM values slightly lower than those of CR, with a mean value of 217 vs 224 g kg^-1^.

**Table 3:** Proportional aggregate weight (g kg^−1^) of sand-free aggregate-size fractions acquired from wet sieving as affected by soil cover and mean weight diameter (MWD). Aggregate-size fraction divided as macroaggregates with large size (> 2 mm, LM) and small size (2 mm—250 μm, sM), microaggregates (250 μm—53 μm, m), and silt and clay (< 53 μm, s + c).

Soil layer	Soil cover	Aggregate-size fraction (g kg^−1^ soil)—Sandfree				MWD (mm)
		LM	sM	m	s + c	
0–10 cm	Bare soil (control)	67g	495a	309abcde	129bcd	0.94hi
	*Trifolium michelianum*	57g	423bc	346a	173a	0.82i
	*Medicago polymorpha*	89fg	462ab	317abcd	133abcd	1.02ghi
	*Medicago lupulina*	148ef	464ab	275def	114cd	1.30defg
	*Medicago truncatula*	66g	477ab	327abc	130abcd	0.92hi
	*Lotus corniculatus*	461a	313de	163g	63e	2.68a
	*Festuca arundinacea*	251bc	339de	263ef	148abcd	1.67bc
	*Festuca ovina*	241bcd	347de	280cdef	132abcd	1.63bcd
	*Festuca rubra commutata*	163def	348de	331ab	158ab	1.26efg
	*Poa pratensis*	210cde	369cd	277def	144abcd	1.51cde
	*Lolium perenne*	219cde	298e	327abc	156abc	1.48cdef
	*Dichondra repens*	211cde	357cde	289bcde	143abcd	1.50cde
	*Trifolium subterraneum*	122fg	439ab	298bcde	141abcd	1.15fgh
	*Sagina subulata*	215cde	366cd	283cde	137abcd	1.53cde
	*Glecoma hederacea*	316b	343de	234f	107d	2.00b
	*Hieracium pilosella*	255bc	367cd	262ef	116bcd	1.73bc
*P-value*		< 0.001	< 0.001	< 0.001	< 0.001	< 0.001
10–20 cm	Bare soil (control)	35e	508a	327ab	130c	0.80bc
	*Trifolium michelianum*	36e	446abcde	338a	180ab	0.74c
	*Medicago polymorpha*	57cde	459abcde	331a	153abc	0.86bc
	*Medicago lupulina*	54cde	431abcde	343a	172abc	0.81bc
	*Medicago truncatula*	58cde	440abcde	353a	149abc	0.84bc
	*Lotus corniculatus*	319a	309f	230b	143bc	1.98a
	*Festuca arundinacea*	109bcd	402bcde	325ab	164abc	1.05bc
	*Festuca ovina*	99bcde	423abcde	318ab	160abc	1.02bc
	*Festuca rubra commutata*	65cde	385def	360a	190a	0.82bc
	*Poa pratensis*	94bcde	491ab	264ab	151abc	1.07bc
	*Lolium perenne*	136b	376ef	333a	156abc	1.16b
	*Dichondra repens*	104bcde	396cdef	323ab	177ab	1.02bc
	*Trifolium subterraneum*	48de	475abc	330a	147abc	0.83bc
	*Sagina subulata*	66cde	468abcd	308ab	159abc	0.91bc
	*Glecoma hederacea*	119bc	389cdef	326ab	166abc	1.09bc
	*Hieracium pilosella*	67cde	442abcde	345a	147abc	0.89bc
*P-value*		< 0.001	< 0.001	0.003	0.003	< 0.001

Lowercase letters indicate differences among treatments within the same soil layer. P-values are reported.

The highest small macroaggregates (sM; 250–2000 µm) in the topsoil layer were found in the bare soil and similarly high values were found in *M. polymorpha* (MP), *M. lupulina* (ML), and *M. truncatula* (MT), while *L. perenne* (LP), with 298 g kg^−1^ had the lowest amount. Within the 0–10 cm soil layer, GR scored the lowest mean sM (340 g kg^−1^), while CR species ranged between 343 (*G. hederacea,* GH) and 439 (*T. subterraneum*, TS) g kg^−1^. The overall range of variation among species within the sM fraction at 0–10 cm was 66% (bare soil vs *L. perenne*) vs. the 707% variation (*L. corniculatus*vs *T. michelianum*,) recorded for the LM fraction (Table 3). Within the upper soil layer, *T. michelianum* (TM) stands out for the highest values for both microaggregates (m, 53–250 µm) and silt and clay fractions (s + c, < 53 µm) recording 346 and 173 g kg^-1^, respectively. Even though belonging to the same group, *L. corniculatus* had the opposite behaviour, recording the lowest values for both m (163 g kg^−1^) and s + c (63 g kg^−1^).

At 10–20 cm soil depth, *L. corniculatus* with 319 g kg^−1^ LM again outscored all other CCs. A quite homogeneous situation could be spotted within GR; measured LM fractions ranging between 65 and 136 g kg^-1^ highlighted GR as the most efficient group in LM production in the lower 10–20 cm depth. *T. michelianum* (TM) is the only one showing an LM value as low as the one of bare soil (36 g kg^-1^).

Within the 10–20 cm soil layer, a more uniform behaviour was found among species for sM, m and s + c under a range of variation of 64% (bare soil vs *L. corniculatus*), 56% (*F. rubra commutata* vs *L. corniculatus*), and 46% (*F. rubra commutata* vs bare soil) respectively vs. the 811% variation (*L. corniculatus* vs bare soil) recorded for the LM fraction (Table 3).

*L. corniculatus* registered the highest mean weight diameter (MWD, mm) among all CCs in both upper (2.68 mm) and lower (1.98 mm) soil layers (Table 3), while *T. michelianum* ranked the lowest (0.92 and 0.74 mm, respectively). Within the first 10 cm, GR showed a more homogeneous pattern with an MWD variability of 32% (*F. rubra commutata* vs *F. arundinacea*), increasing to 73% in CR (*T. subterraneum* vs *G. hederacea*) and 226% in LE (*T. michelianum* vs LC). Similarly, at 10–20 cm depth, the highest variability was registered in LE (167% for *T. michelianum* vs *L. corniculatus* comparison). Conversely, less variability was found within GR (41% for FRC vs *L. perenne* ) and CR (26% for *T. subterraneum* vs *G. hederacea*).

**Figures 3:**
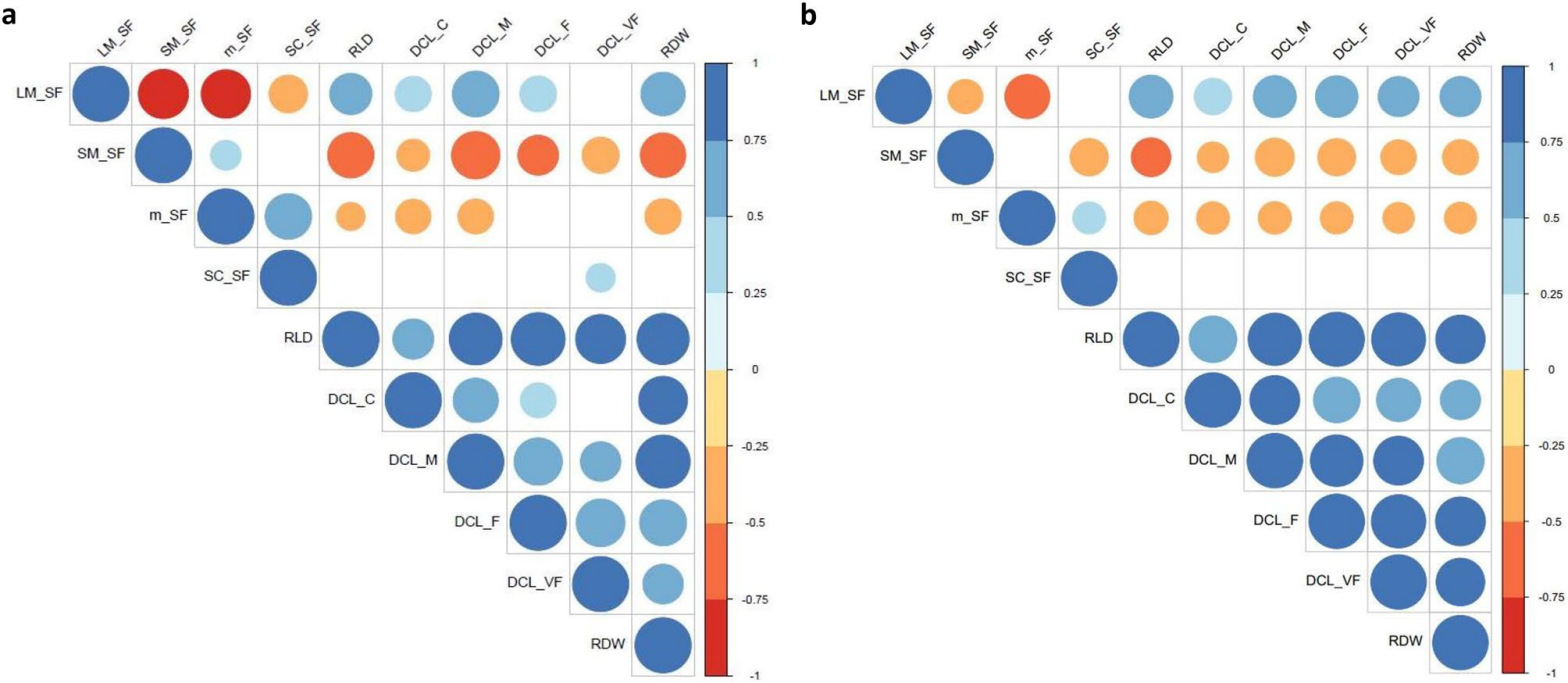
Spearman’s correlations for differences in soil aggregate pattern and root traits for both 0–10 cm (**a**) and 10–20 cm (**b**) soil depth. Blue colour indicates positive correlation, while red indicates negative correlation.

Spearman coefficients (ρ) calculated for the correlations between the aggregate-size fractions, RLD, DCL and RDW are shown in Fig. 3 for the 0–10 cm (A) and 10–20 cm (B) soil depths. For the topsoil layer (Fig. 3a), LM had a close positive correlation with RLD (ρ =  + 0.56), DCL_M (ρ =  + 0.69) and RDW (ρ =  + 0.62). Conversely, sM was negatively correlated with the same diameter class lengths (ρ = − 0.68, − 0.74, and − 0.65, respectively). Overall, a similar pattern was maintained for the 10–20 cm depth, although correlations were in general less tight (Fig. 3b).

### PCA analysis

The Pearson correlation matrix calculated through the Principal Components Analysis (PCA) (Table S1) for the data pool over the 15 CCs showed that evapotranspiration before mowing (UMW_ET) was not correlated to RLD or any DCL; rather, a very close correlation (r = 0.96) was found vs ADW_MW1. Conversely, ET evaluated 25 days after mowing (MW_ET_25) showed a significant positive correlation with several root growth variables including DCL_C, DCL_M, RDW, and total above-ground dry weight (i.e. the sum of first and second cuts, ADW_TOTAL).

**Figure 4:**
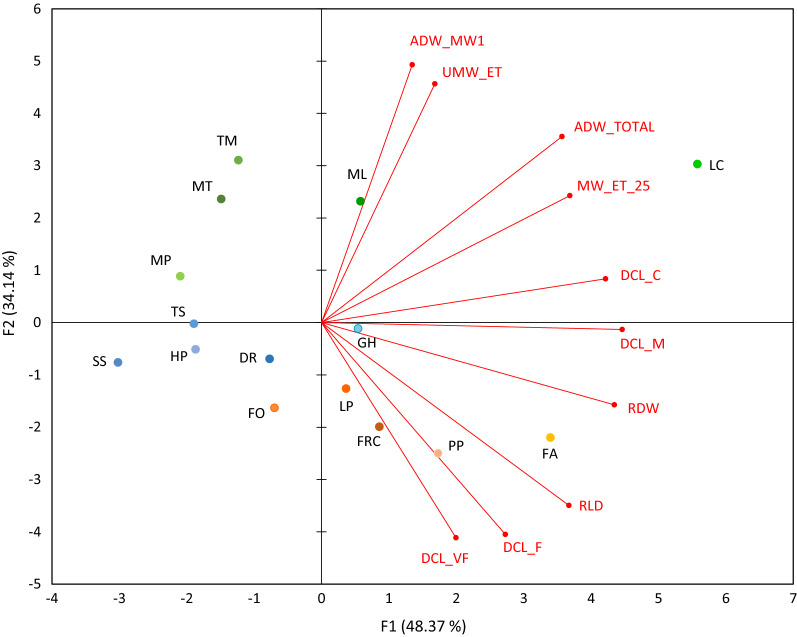
Principal component analysis for 15 different cover crop species divided as grasses (orange shades), legumes (green shades) and creeping plants (blue shades). Red lines represent active variables like (i) evapotranspiration before (UNMW_ET) and (ii) 25 days after mowing (MW_ET_25), (iii) above-ground dry clipped biomass at first mowing event (ADW_MW1) and (iv) total (ADW_TOTAL); (v) diameter class length for very fine (DCL_VF), (vi) fine (DCL_F), (vii) medium (DCL_ M) (viii) coarse (DCL_C) roots; (ix) root length density (RLD) and (x) root dry weight (RDW). Root traits are mean values of 0–20 cm soil depth.

Analysis of the bi-plot (Fig. 4) reporting the positioning of each CC and the direction and magnitude of variation of each variable along F1 and F2 components, enables quite a sharp separation of the three family groups, though with some within-group exceptions.

Within LE, *L. corniculatus* (LC) clearly isolated itself from the remaining species. *L. corniculatus* combined a strong and positive correlation with RDW, DCL_C and DCL_M along the F1 component and with UMW_ET and ADW_MW1 along the F2 component. Conversely, the location of *M. truncatula* (MT), *T. michelianum* (TM) and *M. lupulina* (ML) in the biplot was dependent on a close positive correlation along F2 with UMW_ET and ADW_MW1. *M. polymorpha* (MP) displayed a further distinct behaviour, determined by a strong negative correlation with RDW, DCL_C, DCL_M along the F1 component.

GR grouped in the bottom-right quadrant, except for *F. ovina* (FO). Once again, though, a different behaviour between *F. arundinacea* (FA) and *F. ovina* (FO) was apparent, with the remaining grass species having an intermediate behaviour. *F. arundinacea* showed a close positive correlation with RLD and RDW along F1, and a negative correlation with DCL_F and DCL_VF along F2 (Fig. 4). Conversely, *F. ovina* (FO) has a negative correlation with UMW_ET and ADW_MW1 (F2) and, albeit lower in magnitude, with DCL_M, DCL_C and RDW (F1). The three remaining grass species (*L. perenne*, *F. rubra commutata* and *P. pratense*) were essentially grouped together, albeit their behaviour was driven by negative factor scores along the F2 principal components. These CCs set for a negative correlation with UMW_ET and ADW_MW1 and a positive correlation with DCL_VF and DCL_F.

CR had a somewhat more homogeneous behaviour, although *G. hederacea* (GH) too tended to be isolated in the bi-plot distribution. *S. subulata* (SS), *H. pilosella* (HP) and *T. subterraneum* (TS) were almost insensitive to the variables depicted in F2, whereas their behaviour was largely determined by a negative correlation with some F1 variables, viz., DCL_C, DCL _M and RDW.

## Discussion

The results in the present study shed light on a key issue for the ecological transition of modern viticulture under the threat of a changing climate, viz., how and which CC species should be used at the field level to improve the agro-ecosystem performance.

Although our trial was conducted in pots under inherently constrained conditions and a well-watered regime, the detailed and rich nature of the collected data enables the identification of suitable or less suitable CC species. Even if these CCs were here analyzed with the idea of viticultural implementation, the results obtained can be considered valuable information for application in generic orchards.

Concerning ET, measurements taken after 105 days of undisturbed growth indicated very high rates (around 20 mm day^−1^ under well-watered conditions) in all legumes, with the partial exception of *M. polymorpha* (MP). Taking LC as an example, at the same estimated LAI (about 5), ET was higher than those recorded in New Zealand^42^, peaking at about 11 mm day^−1^. The reasons for this discrepancy are probably related to the significantly lower evaporative demand in their experiment than ours since air temperatures during the central hours did not exceed 21 °C, while our daily temperature ranged between 17 and 29 °C (Fig. S1). Under well-watered conditions and apart from the role played by evaporative demand, daily ET is primarily driven by two factors: the amount of aerial biomass produced and the genetically determined ET_LEAF_. Our results showed that high ET by the LE group mostly derived from a very fast development after sowing, rather than from higher ET_LEAF_, the latter being more than halved compared to the average value in the GR group (Table 1).

While mowing is known to be a valuable tool to limit water consumption by CCs^33^, the availability of previous outcomes quantifying the amount and dynamics of water saving due to the grass cutting is limited. One study found that three weeks after cutting *Medicago sativa* L., daily ET was around 60–70% of that before the cut^43^. Our MW_ET_25 confirms a similar behaviour with ML, while *L. corniculatus* (LC) promptly recovered fully pre-mowing rates (Fig. 1).

Here, for the first time, we found that plotting LAI vs ET reduction for data pooled over the 15 species (Fig. 2b) yielded a very close fit to the observed data, thus suggesting that (i) a linear decrease in ET is expected anytime the LAI is removed through trimming ranges between 0 and 6; (ii) a saturation effect seems to be reached beyond this limit, probably because with a cover canopy increasing in height and density, the bottom leaf layers become heavily shaded, thereby minimising their contribution to transpiration^33^. This has some relevant implications when a temporary winter cover crop, usually containing legumes, is sown for the termination in spring under a green manure purpose. If a desirable feature is obtaining the highest biomass before termination to maximise the N return to the crop, a legume growth above an LAI of 6–7 will not cause luxury water use, based on the mechanism highlighted above.

High water use by *L. corniculatus* (LC) was corroborated by the highest RLD in the 10–20 cm soil layer (7.9 cm cm^−3^) and RDW in both the 0–10 cm (8.7 mg cm^−3^) and 10–20 cm (4.5 mg cm^−3^) depths. However, our results also clarify that such an effect is primarily due to the very high values of DCL_M and DCL_C, which in turn explain why *L. corniculatus* (LC) was also able to dig into the lower soil layer. Since thicker roots have been reported to be more effective at overcoming issues related to soil mechanical resistance^44^, our results also suggest the importance of *L. corniculatus* (LC) in improving physical soil quality by decreasing bulk density and preventing soil compaction, even though tillage operations are suspended.

Turning to GR, our results add significant knowledge to previous studies^45–47^ leading to the potential use of grass-based permanent mid-row CCs in orchard floor management. The present trial indicates that *F. arundinacea* (at least as far as the tested cultivar) has to be regarded as quite competitive grass, while *F. ovina* behaves contrarily. The transpiration potential of *F. arundinacea* relies more on high ET_LEAF_ rather than on fostered aerial biomass: hence the ability for higher light interception (Fig. 1 and Table 1). This is substantially different from what was reported above on the LE group (especially *L. corniculatus*) and suggests a relevant implication for the use at the field level: at the same LAI, any of our tested GR species will probably use a significantly higher amount of water than legume species.

Our results on GR ET are confirmed by the literature, where *F. arundinacea* and *F. ovina* are assessed as the most and the least competitive grasses, with values as high as 8.5 and 12.6 mm day^-1^, respectively^45,48^. Moreover, one study^46^ showed the existing difference between *F. arundinacea* cultivars grown under non-limiting water nutrient conditions whose ET rates ranged between 10 and 13.5 mm day^-1^, perfectly fitting our data. Inputting our *F. arundinacea* mowed values (161.8 g m^-2^) in an ET reduction vs dry clipped biomass model made on *Festuca arundinacea* var. Barfelix^33^ leads to a 36% ET reduction, which is a very close fit to the 35% reduction registered at an LAI of 1.52 (Table 1 and Fig. 2b).

Notably, while *L. corniculatus* (LC) and *F. arundinacea* (FA) share the capacity to spread their roots into the deeper soil layer, our PCA analysis revealed that values of DCL for any root diameter – thus including very fine and fine roots – under *F. arundinacea* in the 10–20 cm soil layer were several times higher than those under other grasses. It is widely accepted that a well-established and developed root system is essential for the efficient absorption of water^49^. Therefore, our results on DCL indicate that *F. arundinacea* can further enhance the absorption of nutrients and water too, by increasing the root hair surface even in the lower soil layers.

All tested GR species, despite large differences in root growth parameters, retained high and similar ET_LEAF_ values. Explanation of such a behaviour is found in ET rates given on a pot basis (Fig. S2). It is quite striking that for any GR, daily pot ET stayed within 60% of the daily water supply (1.0–1.1 L per pot). Presumably, this allowed optimal leaf function, explaining why ET_LEAF_ did not differ. Consequently, under persisting non-limiting soil water availability, total water use in our tested GR becomes a primary function of LAI (Fig. 2b).

FO confirms its attitude to low ET due to its “dwarfing” characteristics. A very low ADW_MW1 (Table 1) associated with a shallow root system with minimum soil colonization below 10 cm depth renders this CC a quite interesting candidate for a permanent between row establishments. According to the PCA analysis, *F. ovina* (FO) isolated for a negative correlation with ADW_MW1 and UMW_ET. Ideally, in the field, its shallow root system might facilitate temporal and partial drying in summer, with a prompt recovery with incoming precipitation in the fall.

Turning to the under-vine strip management, a lot of work has been done to investigate how native vs sowing of commercial mixtures might affect the degree of competition towards the root system of the consociated vines (essentially insisting within the same soil volume) with possible effects on the RLD and root distribution^50^. Several authors have found that the topsoil layer conquered by a CC will induce the grapevine root system to explore deeper soil horizon or to preferentially spread sideways from the row axis^50–52^. Previously conducted research^53^ showed that well-established grapevines with understory *F. rubra* grass grew a deeper root distribution and showed a little evidence of restricted-water uptake. Only at 10 cm soil depth the O ^18^O isotope depletion (δ^18^O, ‰) was significantly more negative in the soil with the CC relative to the tilled one, but there was no significant treatment effect below that.

Preliminary work conducted in France^40,41^ has shown that establishing shallow-rooted, yet creeping and smothering CCs under the row strip can be quite successful at controlling weeds, thereby reducing the need for tillage or herbicides. At the same time, the grapevine root system will grow underneath the CC, where higher soil moisture is likely to be available. The management of such CCs implies that no mowing is made until the cover outgrows and tends to invade the alley. Therefore, in our trial, we avoided any canopy-shortening cut until the cover started to overflow the pot surface. Such a status was reached by *G. hederacea* (GH) and *T. subterraneum* (TS) only. ET reported in Fig. 1 strongly supported the assumption that all CR species retain good water-saving characteristics and for three of them (DR, HP and SS), pre-mowing ET rates were slightly lower than those measured on the control and less than 400 g H_2_0 pot^−1^ day^−1^ were used (Fig. S2). Despite the actual lack of mowing, data taken on these species at 17 and 25 days after mowing showed a mild increase in ET rates that have to be inherently attributed to a likely thickening of the CC within the pot surface. The second feature which was likewise shared by all CR was that root colonization was essentially restricted to the topsoil layer only (Table 2), thus obeying the need of having, under field conditions, two well-separated soil layers, including grass roots on top and grape roots at higher depths.

In our study, a careful assessment of CCs’ effects on soil aggregate stability and MWD was performed and associated to root traits. It is well known that soil aggregate size and stability are positively associated with infiltration (and retention) of water and mitigation of soil erosion, due to improved pore size distribution^54^. In addition, LM plays a major role in enhancing SOM concentration and stabilization^55^, thus further increasing water and nutrient availability for the cultivated plants. Indeed, it is well known that macro-aggregates provide physical protection to SOM by binding organic compounds to soil minerals and creating a barrier between microorganisms and their substrate^56^. Since Mediterranean vineyards are usually established on steep slopes^57^, our study shows that selected CCs may be considered a promising tool to boost soil aggregation, thus suggesting increased water infiltration, as well as reduced soil erosion and nutrient losses^58^. As soil water evaporation is mainly affected by soil water content, organic matter, texture and structure^59^, it is reasonable to assume that CCs may contribute to its change with different magnitude depending on the species characteristics. However, in our trial the evapotranspiration components (i.e. evaporation and transpiration) could not be distinguished, and the different cover crop-induced-soil aggregation effect on soil evaporation was hard to assess as water loss measurements were conducted a few months before the soil sampling and the following aggregate determinations. More in general, little information seems available: increased aggregate stabilization was assessed to increase the amount of water available in soils for plants by reducing losses via evaporation^60^, whereas a more recent work has shown no significant impact of soil particle size on evaporation rate^61^.

Our results also show a positive correlation between large macro-aggregates and roots development parameters, such as RLD and RDW, thus suggesting that roots are the main drivers of soil aggregate formation and stabilization in this system. Indeed, roots are known to produce mucilage and other exudates that hold particles together, hence promoting LM formation^62^. Similar results were reported in the previous studies^63–65^, which observed a positive correlation between root biomass/length density and aggregate stability. Therefore, CCs with high RLD and RDW should be suggested to promote aggregate stabilization, increasing soil organic carbon (SOC) protection and water infiltration/retention.

In our study, GR generally enhanced RLD and RDW, compared to LE and CR plants, except for *L. corniculatus* and *G. hederacea*. In particular, *F. arundinacea* showed the highest RLD (59.0 cm cm^-3^) among all species and one of the highest LM contents (251 g kg^-1^ soil) in the 0–10 cm soil layer, thus confirming the positive interaction between RLD and LM stabilization. Among legumes, *L. corniculatus* had the highest amount of LM in both soil layers, establishing itself as a promising CC for improving soil structure, while being an external source of N due to N-fixation. This may be explained by the higher DCL (Ø > 1.0 mm) of *L. corniculatus* compared to other species, thus indicating the important role of large roots in soil aggregation levels. The strong influence of *L. corniculatus* on soil strength, when grown in monocultures compared to other legumes, has been reported. Interestingly, *H. pilosella* had higher LM compared to most of the other species, while having lower RLD and RDW. Previous studies reported lower pH of soil under *H. pilosella* than under other plants^66,67^, which was found in turn to be negatively correlated with water stable aggregates^68^. The authors explained the negative correlation between the pH increase and the soil aggregation level by the higher loading of humic acids on the mineral surfaces and by a decrease in the electrostatic repulsive forces between negatively charged substances under soil acidic conditions, resulting in higher coagulation of organic and mineral particles. Therefore, for *H. pilosella*, the effect on soil aggregation is more related to changes in soil chemical properties rather than to root characteristics.

## Conclusions

The current pot trial is, to the best of our knowledge, the first case of a comparative screening to evaluate the water use, root characterization, and soil-aggregation potential of a large number of herbaceous species and define the most recommended ones for vineyard usage. The highest ET rates recorded for legumes were mainly due to a very fast development after sowing, rather than to a higher ET_LEAF_. For both legumes and grasses, mowing was confirmed as a valuable practice to limit water use proportionally anytime until a LAI of 5–6, while a saturation effect seems to be reached beyond this limit. Among grasses, *F. ovina* was assessed to be the one with the lowest ET, which renders it an interesting candidate as a permanent between-row living mulch. Moreover, ideally, once used in the field, its shallow root system might facilitate temporal and partial drying in summer, with a prompt recovery with incoming rainfall in the fall. CR confirmed their potential for under-trellis strip management as, while maintaining a full soil coverage (i.e. potentially successful in weed control), they did not need any mowing for height reduction, registered low water use rates and a superficial (i.e. 0–10 cm) root colonization. Lastly, our study showed that CCs with enhanced RLD and RDW such as GR, *G. hederacea* and *L. corniculatus* may be considered promising species to boost soil aggregation, increase SOC protection and water infiltration, as well as reduce soil erosion and nutrient losses.

## Materials and methods

### Plant material and experimental layout

The study was conducted in 2020 at the Department of Sustainable Crop Production, Università Cattolica del Sacro Cuore (Piacenza, Northern Italy, 45°2’ N; 09°42’ E) on 64 pots of 15 L volume (0.27 m deep, with an internal diameter of 0.27 m) kept outdoor in a pot-lot. Pots were filled with clay-loam soil having 35% sand, 36% silt and 29% clay. Field capacity, permanent wilting point, and soil bulk density were estimated at 32.8%, 18.7% and 1.42 g cm^-3^, respectively^69^.

The experiment was set up as a randomized complete block design (RCBD) with four replicates and sixteen treatments: a control (i.e. bare soil) and fifteen CCs, which were tested as divided into three groups: (i) grasses, (ii) legumes, and (iii) creeping plants (Table S2).

CCs’ sowing rate was computed according to a previous germination test (Table S2) and all CCs were manually seeded on 20 April 2020. By the time the first measurements were made, they all had 100% soil coverage and no weed growth was recorded. To aid plant establishment and avoid any water deficit, throughout the trial period, each pot was supplied with 350 mL of water three times per day (i.e. 55% of total available water) delivered by an automated single dripper. Automated watering was stopped a day before ET measurements and the exact amount of 1 L pot^-1^ was given manually.

During the trial season, pot management consisted of two mowing events and two tillage operations. On 6 July and 20 September 2020, grasses and legumes were hand-mowed to ~ 4 cm above soil surface while creeping plants were only trimmed, as it concerns the aerial biomass exceeding the pot’s edges, as they are not supposed to be trimmed in height under open field conditions. On the same days, light soil tillage (around 3 cm depth) was performed in the bare soil pots using a three-tooth rake.

### Evapotranspiration measurements, above-ground biomass and roots sampling

CCs’ ET measurement was performed through a gravimetric method as, at each measuring date, all pots were weighed at 8 a.m. and 7 p.m. with an electronic scale with a resolution of 0.01 g. The daily CC ET (mm d^-1^) was calculated as ΔW/S, where ΔW is the change in the pot mass between the 2 daily weights, and S is the surface area of the pot^33^. ET rates measured were then referred to per square meter of removed leaf area (ET_LEAF_) when needed. Daily maximum, mean and minimum air temperature (°C), together with daily precipitation (mm), were monitored throughout the experiment, and data were collected from an automated meteorological station positioned next to the experiment pot-lot (Fig. S1).

On 6 July and 20 September 2020, the hand-cut biomass was collected and placed in a ventilated oven at 105 °C until constant weight and then the above-ground dry weight was measured as first (ADW_MW1) and second (ADW_MW2) mowing. Total above-ground dry weight (ADW_TOTAL) was calculated as the sum of the two cuts.

Before mowing, the above-ground fresh biomass of 20 plants from each tested CC (i.e. five plants per pot) was sampled and the equivalent leaf area was measured using the image-analysis Image J software (National Institutes of Health, Bethesda, MD, USA)^70^. The sampled biomass was then dried in a ventilated oven at 105 °C until constant weight. Cover crop LAI on the first day of ET measurement was estimated fitting the ADW_MW1 in the linear regression leaf area vs dry weight linear regression obtained for all CC tested (Table S3).

For CR alone, as the above-ground biomass was clipped exclusively when exceeding the pot borders, LAI was estimated through pot photo-analysis. The total leaf number per pot was counted on the photo prints (despite the surface pot being completely covered, leaves were clearly visible as just a few overlaps occurred) and multiplied by CC mean leaf area (known from the leaf sampling mentioned above).

Root sampling was conducted on September 29 with a self-constructed “Shelby” tube sampler of known volume (6.88 cm diameter and 23.2 cm length) that was inserted into the soil to reach 0.2 m depth. Soil samples for each pot were taken at an intermediate position between the edges and the centre of the pot. Each soil core was divided into two layers: 0–10 cm and 10–20 cm soil depths. Two more samples per pot were then taken at the end of the trial, on 4 February 2021, with a tubular soil sampler (2.5 cm diameter) for aggregate stability analysis. The litter (if present) was removed, and each soil core was divided into 0–10 and 10–20 cm depths. Soil samples were passed through an 8 mm sieve through gentle breaking^71^, air-dried and stored at room temperature for subsequent determinations.

### Root characterization

Soil cores were stored at − 20 °C until root separation and analysis were carried out. After defrosting, samples were kept in a solution of oxalic acid (2%) for 2 h to facilitate the separation of roots from soil^72^. Soil samples were then washed and cleaned. The roots were recovered from the water using a 2 mm sieve^72^. Finally, the roots were hand-cleaned from organic particles, immersed in 10% (v/v) ethanol solution^73^ and stored at + 4 °C. For scanning, roots were placed on a transparent plastic tray. Distilled water was added to the tray to facilitate the layout of the root and minimise overlapping.

The roots’ images were acquired by a scanner (Epson Expression 10000xl, 600 dpi) equipped with a double light source to avoid root overlapping^74^. The software WinRHIZO Reg 2012 (Regent Instrument Inc., Quebec, Canada) was used to determine RLD (cm cm^-3^) and the root diameter (RD, mm). RLD within each diameter class – namely the DCL (mm cm^-3^) – was calculated for very fine (DCL_VF, < 0.075 mm), fine (DCL_F, 0.075–0.2 mm), medium (DCL_M, 0.2–1.0 mm) and coarse (DCL_C, > 1.0 mm) roots, as adapted from Reinhardt and Miller^75^. Moreover, RDW (mg cm^-3^) was gravimetrically determined after drying the roots in a ventilated oven at 60 °C until constant weight.

### Soil aggregate distribution and mean weight diameter

Subsamples of 80 g were dipped into deionized water for 5 min and wet sieved. Three sieves of 2000 µm, 250 µm, and 53 µm meshes were used to separate the four aggregate fractions: LM (> 2000 µm), sM (250–2000 µm), m (53–250 µm) and s + c (< 53 µm). Each fraction was isolated by manually moving the sieve up and down 50 times. After each phase, soil aggregates remaining on the top of the sieve were transferred onto an aluminium pan, oven dried at 105 °C and weighed. Water and soil passing through the sieve were poured onto the smaller sieve mesh, thus starting the next phase (wet-sieving). All fractions were corrected for sand content, and the MWD was calculated according to van Bavel^76^ as follows:

1 $$MWD= \sum_{i=1}^{n}{x}_{i}{w}_{i},$$

where ${x}_{i}$ is the mean diameter of each aggregate-size fraction separated by sieving, and ${w}_{i}$ is the proportion of each sand-free aggregate-size fraction out of the entire sample weight.

### Statistical analysis

All data were subjected to a one-way analysis of variance (ANOVA) using IBM SPSS Statistics 27 (SPSS Inc., Chicago, USA). In case of significance of the Fisher test, mean separation was performed through the Student-Newman Keuls (SNK) test (p < 0.05).

Principal Component Analysis (PCA) was also carried out on 10 representative variables of both below and above-ground growth (RLD, DCL_VF, DCL_F, DCL_M, DCL_C, RDW, ADW_MW1, and ADW_TOTAL) and water use (UMW_ET and MW_ET_25) using the XLSTAT statistical package (Addinsoft, New York, NY, United States). The chosen PCA was a Pearson correlation matrix. The number of filter factors was set at 5 and the final data visualization was in the form of a distance bi-plot.

A correlation analysis was performed separately for the two soil depths considered (0–10 and 10–20 cm) to assess the relationship between root traits (RLD, DCL_VF, DCL_F, DCL_M, DCL_C, RDW) and aggregate size fractions (LM, sM, m, s + c, MWD), using the non-parametric Spearman rank coefficient (ρ). A p-value of 0.05 was considered significant for the test. We used R 4.0.3.^77^ with factoextra^78^ package for the Spearman’s rank correlations, respectively.

### Ethical approval

Experimental research and pot studies on the cultivated plants, including the collection of plant material here reported, comply with relevant institutional, national, and international guidelines and legislation. Moreover, plant specimens collection was made in line with the appropriate permissions.

## Supplementary Information

Supplementary Information.

## Data Availability

All data generated or analyzed during this study are included in this published article and its Supplementary Information files.
