# Supplementary material for: A comparative study of fifteen cover crop species for orchard soil management: water uptake, root density traits and soil aggregate stability
Source: Sci Rep. 2023 Jan 13;13:721. doi: 10.1038/s41598-023-27915-7 (PMC9839681; doi:10.1038/s41598-023-27915-7)
Supplement: Supplementary file 1 — Supplementary Information. [file 41598_2023_27915_MOESM1_ESM.pdf]

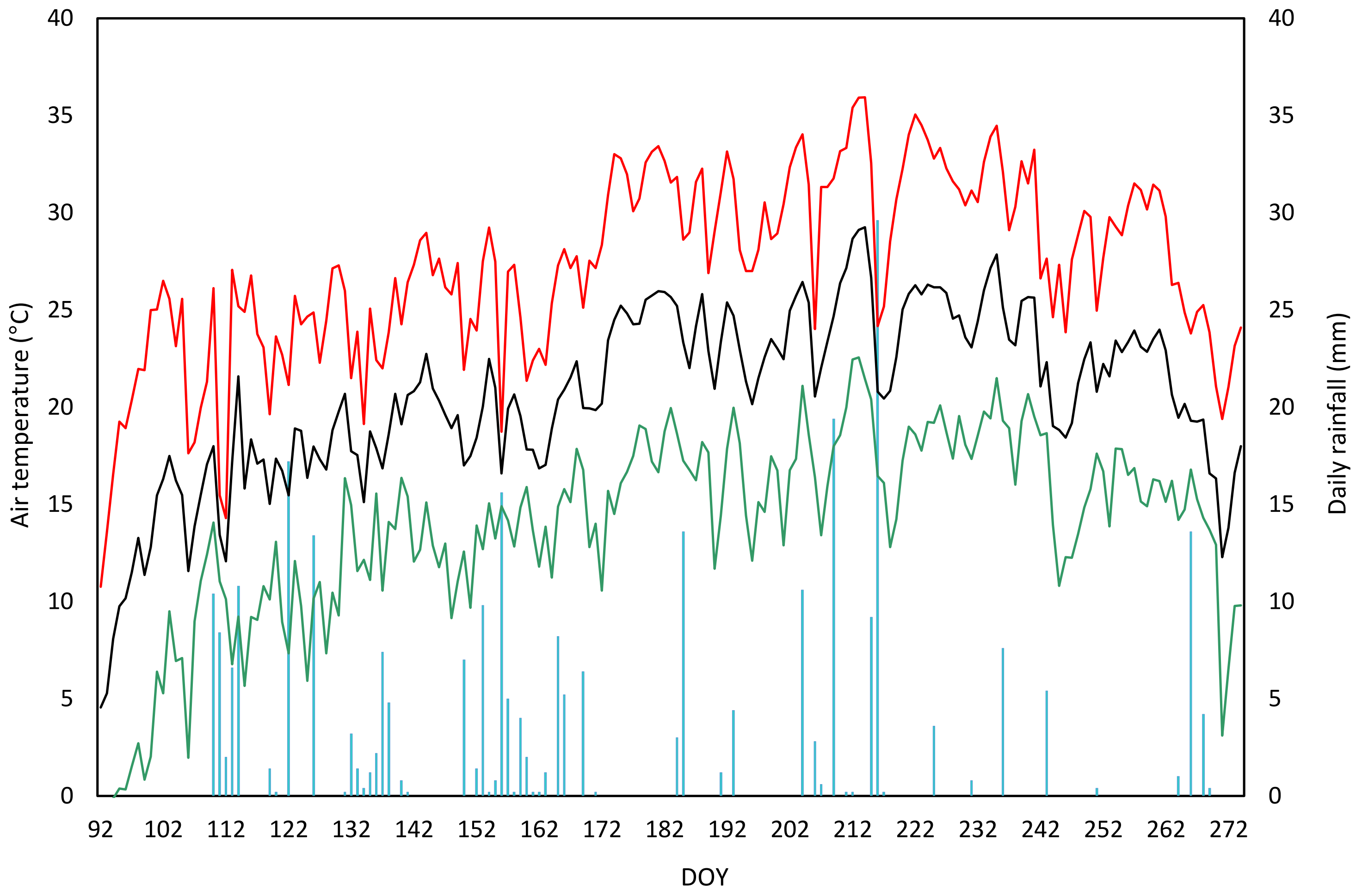


Figure S1. Daily maximum (red line), mean (black line), and minimum (green line) air temperature for 1 April to 30 September in 2020 at a weather station located within the experiment site. Histogram shows daily precipitation. DOY, day of the year.

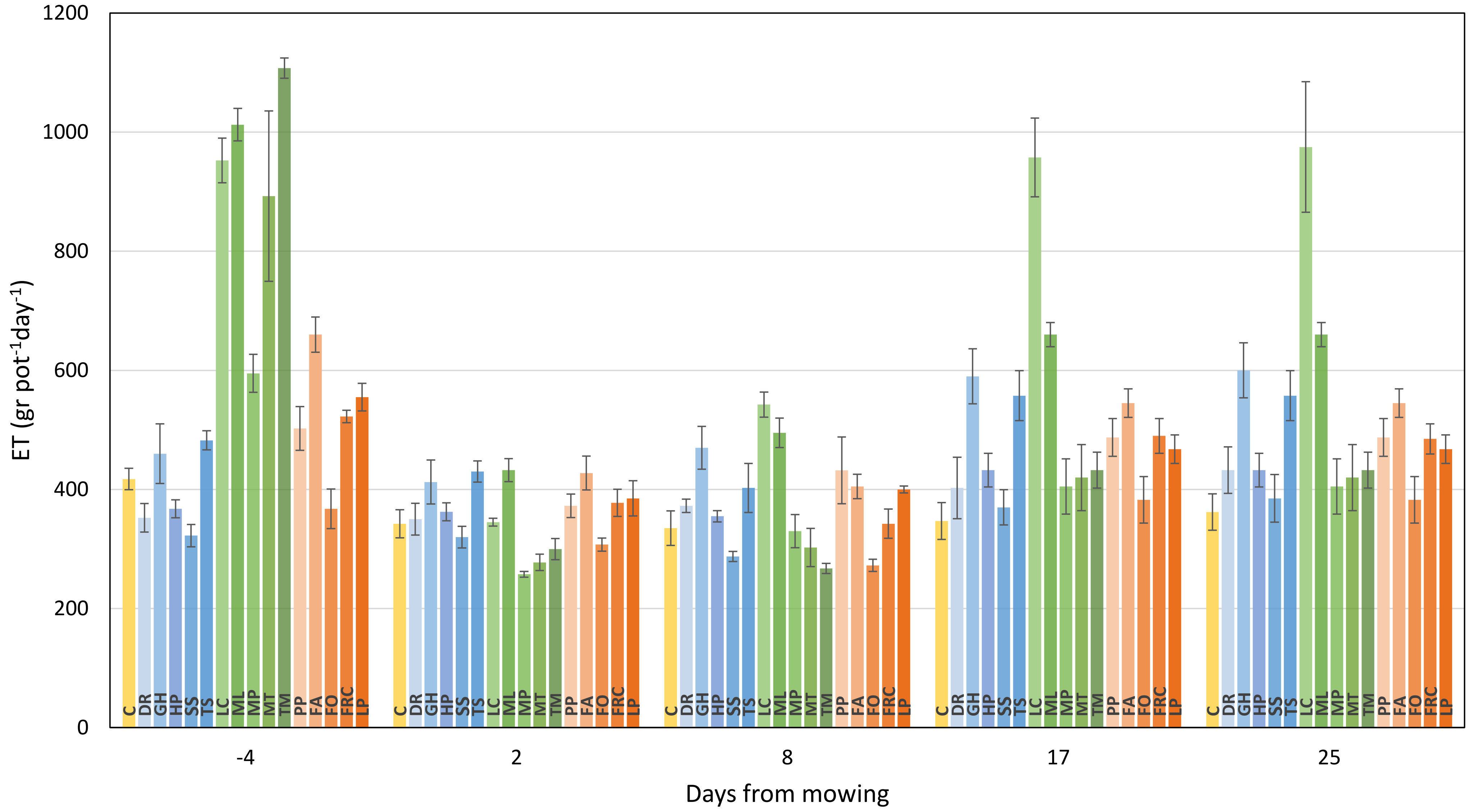


Figure S2. Vertical bars represent the daily water use as referred to the pot surface (ET, g pot$^{-1}$ day$^{-1}$) for the bare soil (yellow) and all the cover crop species, divided into creeping plants (shades of blue), legumes (shades of green) and grasses (shades of orange). Evapotranspiration was measured though a gravimetric method before (i.e., -4) and at 2, 8, 17 and 25 days after mowing. ET data are mean values ± SE (n = 4).

Table S1. The Pearson correlation matrix calculated through the PCA analysis for data pool over the 15 cover crop tested on 10 representative variables is shown here. Variables are reported as following: root length density (RLD), diameter class length for coarse (DCL_C), medium (DCL_M), fine (DCL_F), and very fine (DCL_VF) roots, root dry weight (RDW), above-ground dry biomass clipped at first mowing event (ADW MW1), total above-ground clipped biomass (ADW_TOTAL) and evapotranspiration rates before (UMW_ET) and after (MW_ET_25) grass trimming.

| Variables | RLD | DCL_C | DCL_M | DCL_F | DCL_VF | RDW | UMW_ET | MW_ET_25 | ADW_MW1 | ADW_TOTAL |
|---|---|---|---|---|---|---|---|---|---|---|
| RLD | **1** | 0.513 | **0.665** | **0.917** | **0.860** | **0.802** | -0.123 | 0.288 | -0.224 | 0.195 |
| DCL_C | 0.513 | **1** | **0.932** | 0.218 | 0.164 | **0.782** | 0.274 | **0.679** | 0.276 | **0.693** |
| DCL_M | **0.665** | **0.932** | **1** | 0.423 | 0.261 | **0.884** | 0.164 | **0.659** | 0.150 | **0.652** |
| DCL_F | **0.917** | 0.218 | 0.423 | **1** | **0.799** | **0.669** | -0.226 | 0.174 | -0.359 | 0.005 |
| DCL_VF | **0.860** | 0.164 | 0.261 | **0.799** | **1** | 0.446 | -0.213 | -0.075 | -0.306 | -0.122 |
| RDW | **0.802** | **0.782** | **0.884** | **0.669** | 0.446 | **1** | 0.036 | **0.562** | -0.042 | 0.476 |
| UMW_ET | -0.123 | 0.274 | 0.164 | -0.226 | -0.213 | 0.036 | **1** | **0.545** | **0.960** | **0.729** |
| MW_ET_25 | 0.288 | **0.679** | **0.659** | 0.174 | -0.075 | **0.562** | **0.545** | **1** | 0.447 | **0.834** |
| ADW_MW1 | -0.224 | 0.276 | 0.150 | -0.359 | -0.306 | -0.042 | **0.960** | 0.447 | **1** | **0.748** |
| ADW_TOTAL | 0.195 | **0.693** | **0.652** | 0.005 | -0.122 | 0.476 | **0.729** | **0.834** | **0.748** | **1** |

Values in bold are different from 0 with a significance level alpha=0.05

Table S2. Cover crop tested are here shown as divided into the three groups defined. The seeding rate (g $m^{-2}$) was defined according to the label guidelines of each species and computed according to the germination test. Total above-ground dry clipped biomass (ADW_TOTAL; g $m^{-2}$) -i.e. the equivalent of the two mowings made.

| Cover crop group | Cover crop | Seeding rate (g $m^{-2}$) | ADW_TOTAL (g $m^{-2}$) |
|---|---|---|---|
| Legumes | *Trifolium michelianum* Savi cv. Bolta | 0.96 | 937.65 |
| | *Medicago polymorpha* L. cv. Scimitar | 1.20 | 428.17 |
| | *Medicago lupulina* L. cv. Virgo | 4.20 | 1087.24 |
| | *Medicago truncatula* Gaertn. cv. Paraggio | 1.73 | 780.86 |
| | *Lotus corniculatus* L. cv. Leo | 1.56 | 2121.11 |
| Grasses | *Festuca arundinacea* Schreb. cv. Thor | 38.50 | 630.41 |
| | *Festuca ovina* L. cv. Ridu | 24.00 | 277.20 |
| | *Festuca rubra* L. var. *commutata* Gaud. cv. Casanova | 24.00 | 447.85 |
| | *Poa pratensis* L. cv. Tetris | 8.40 | 598.82 |
| | *Lolium perenne* L. cv. Playfast | 7.00 | 352.86 |
| Creeping | *Glecoma hederacea* L. | 2.92 | 1035.25 |
| | *Hieracium pilosella* L. | 0.73 | 102.80 |
| | *Dichondra repens* J.R.Forst. & G.Forst | 4.00 | 218.11 |
| | *Sagina subulata* (Swartz) C. Presl | 0.07 | 0.00 |
| | *Trifolium subterraneum* L. cv. Denmark | 3.24 | 23.21 |

Table S3. Leaf area ($cm^2$) vs dry weight (g) linear regression and $R^2$ for each cover crop tested.

| **Cover crop group** | **Cover crop** | **Linear regression** | **$R^2$** |
|---|---|---|---|
| Legumes | *Trifolium michelianum* Savi cv. Bolta | y = 353.43x + 0.3896 | 0.89 |
| | *Medicago polymorpha* L. cv. Scimitar | y = 191.1x + 0.9518 | 0.46 |
| | *Medicago lupulina* L. cv. Virgo | y = 215.49x + 0.539 | 0.67 |
| | *Medicago truncatula* Gaertn. cv. Paraggio | y = 177.78x + 0.6999 | 0.84 |
| | *Lotus corniculatus* L. cv. Leo | y = 171.41x + 0.2284 | 0.80 |
| Grasses | *Festuca arundinacea* Schreb. cv. Thor | y = 94.141x + 0.4729 | 0.73 |
| | *Festuca ovina* L. cv. Ridu | y = 199.78x + 0.0821 | 0.53 |
| | *Festuca rubra* L. var. *commutata* Gaud. cv. Casanova | y = 99.508x + 0.3949 | 0.69 |
| | *Poa pratensis* L. cv. Tetris | y = 116.24x + 0.2004 | 0.83 |
| | *Lolium perenne* L. cv. Playfast | y = 114.93x + 0.3919 | 0.72 |
| Creeping | *Glecoma hederacea* L. | y = 199.06x + 1.2761 | 0.74 |
| | *Hieracium pilosella* L. | y = 202.01x + 1.1024 | 0.85 |
| | *Dichondra repens* J.R.Forst. & G.Forst | y = 178.36x + 0.23 | 0.92 |
| | *Trifolium subterraneum* L. cv. Denmark | y = 130.35x + 2.4772 | 0.37 |
